# Small pituitary volume and central nervous system anomalies in Fanconi Anemia

**DOI:** 10.3389/fendo.2024.1385650

**Published:** 2024-08-19

**Authors:** Beatriz Corredor, Inés Solís, Josune Zubicaray, Julián Sevilla, Jesús Argente

**Affiliations:** ^1^ Department of Pediatrics, Hospital Infantil Universitario Niño Jesús, Madrid, Spain; ^2^ Department of Pediatric Endocrinology, Hospital Infantil Universitario Niño Jesús, Madrid, Spain; ^3^ Department of Pediatrics, Hospital Universitario de Toledo, Toledo, Spain; ^4^ Department of Pediatric Endocrinology, Hospital Universitario de Toledo, Toledo, Spain; ^5^ Department of Pediatric Radiology, Hospital Infantil Universitario Niño Jesús, Madrid, Spain; ^6^ Department of Pediatric Hematology, Hospital Infantil Universitario Niño Jesús, Madrid, Spain; ^7^ Fundación de Investigación del Hospital Infantil Universitario Niño Jesús, Madrid, Spain; ^8^ Center for Biomedical Research on Rare Diseases Network (CIBERER), Madrid, Spain; ^9^ Department of Pediatric Endocrinology, La Princesa Research Institute, Madrid, Spain; ^10^ Department of Pediatrics, Universidad Autónoma de Madrid, Madrid, Spain; ^11^ Centro de Investigación Biomédica en Red de Fisiopatología de la Obesidad y Nutriciόn (CIBEROBN), Instituto de Salud Carlos III, Madrid, Spain; ^12^ IMDEA, Food Institute, CEIUAM+CSI, Madrid, Spain

**Keywords:** pituitary gland, pituitary volume, Fanconi Anemia, magnetic resonance imaging, short stature

## Abstract

**Introduction:**

Fanconi anemia (FA) is a genomic instability disorder associated with congenital abnormalities, including short stature and the presence of central nervous system anomalies, especially in the hypothalamic-pituitary area. Thus, differences in pituitary size could associate with the short stature observed in these patients. Our aim was to evaluate whether central nervous system abnormalities and pituitary gland volume correlate with height and hormone deficiencies in these patients.

**Methods:**

In this cross-sectional exploratory study 21 patients diagnosed with FA between 2017 and 2022 in a Spanish Reference Center were investigated. Magnetic resonance imaging (MRI) was performed and pituitary volume calculated and corelated with height and other endocrine parameters.

**Results:**

The percentage of abnormalities in our series was 81%, with a small pituitary (pituitary volume less than 1 SD) being the most frequent, followed by Chiari malformation type 1. The median value of pituitary volume was -1.03 SD (*IQR*: -1.56, -0.36). Short stature was found in 66.7% [CI95% 43-85.4]. Total volume (mm^3)^ increases significantly with age and in pubertal stages. There were no differences between volume SD and pubertal stage, or the presence of endocrine deficiencies. No correlations were found between pituitary volume and the presence of short stature. The intraclass correlation index (ICC) average for volume was 0.85 [CI95% 0.61-0.94] indicating a good‐to‐excellent correlation of measurements.

**Discussion:**

Central nervous system anomalies are part of the FA phenotype, the most frequent after pituitary hypoplasia being posterior fossa abnormalities, which may have clinical repercussions in the patient. It is therefore necessary to identify those who could be candidates for neurosurgical intervention. The size of the pituitary gland is smaller in these patients, but this does not seem to be related to hormone deficiency and short stature or exposure to a low dose of total body irradiation.

## 1 Introduction

Fanconi anemia (FA) is a genomic instability disorder associated with congenital abnormalities, bone marrow failure and cancer predisposition. At least 23 genes have been discovered to play a role in the FA pathway. All pathogenic variants in these genes are autosomal recessive, except FANC-B, which is x-linked, and FANCR/RAD51, which is autosomal dominant. FA proteins are expressed in every tissue with loss of this pathway resulting in organ specific consequences. A clinical hallmark of FA is progressive bone marrow failure (1, 2). Congenital anomalies commonly seen in FA are included in the VACTERL-H association (vertebral abnormalities, anal atresia, cardiac abnormalities, tracheo-esophageal fistula, esophageal or duodenal atresia, renal abnormalities, upper limb abnormalities and hydrocephalus). Six additional common FA features are also grouped into the acronym PHENOS (skin pigmentation abnormalities, small head, small eyes, structural central nervous system abnormalities, otologic abnormalities and short stature). A recent review indicated that that at least 90% of these patients had at the very least one physical feature, with the most frequent being skin pigmentation changes, short stature, upper limb radial abnormalities, small eyes, renal malformations and central nervous system (CNS) findings (3).

Structural anomalies of the CNS, especially of the hypothalamic-pituitary area, have been reported in studies hypothesizing that the etiology of the short stature is associated with a small pituitary gland. Previous studies indicate that in patients with FA the pituitary is smaller based on its height; however, other measurements were not taken into consideration (4–8).

The purpose of this study was to investigate the prevalence of abnormalities found by brain magnetic resonance imaging (MRI), and to perform pituitary gland measurements especially the determination of its pituitary volume in a cohort of FA patients. Moreover, these abnormalities were correlated with clinical and demographic data.

## 2 Material and methods

### 2.1 Patient population

A cross-sectional exploratory study was carried out in pediatric patients at the Spanish Fanconi Anemia reference center. Thirty-eight genetically diagnosed patients have been assessed at this center MRI performed in 21 patients between 2017 and 2022. All MRIs were performed as routine clinical work-up of children with Fanconi Anemia. In all cases, informed written parental consent was obtained to participate in the registry of pathologies affecting growth. It was reviewed and approved by the Ethical Committee of the University Hospital Niño Jesús (n. R-0017/19 and n. R-0069/23). The parents or guardians were informed about the registry by their pediatricians and asked for their written consent. Parents or children/adolescents had the opportunity to withdraw their consent at any time which leads to complete deletion of all data. The study was conducted ethically in accordance with the World Medical Association Declaration of Helsinki.

Demographic (sex, date of birth) and clinical features (FA genotype, hematopoietic stem cell transplantation, radiotherapy) were recorded. Patients with FA were clinically evaluated by the endocrinology department where body weight and up to two time separated standing height measurements were performed. Pubertal status was assessed by Tanner staging. To make comparisons, they were divided into two groups. The prepubertal group were those with Tanner stage I and the pubertal group were those with Tanner stages II, III, IV or V. Laboratory evaluation included: IGF-1 and IGFBP-3 measurements, and a GH stimulation test was performed in case of suspected GH deficiency. Morning cortisol levels and ACTH stimulation tests were performed in suspected cases of deficiency, TSH and free T4 levels were measured, and reproductive hormone evaluation was carried out, including testosterone, estradiol, FSH and LH studies, with an and LHRH test performed in suspected cases of pubertal pathology.

### 2.2 Radiological measurements

Pituitary size was measured using either a thin-section three-dimensional turbo-spin echo T1 weighted sequence MRI with multiplanar reformatted images, or 2 mm thickness sagittal and coronal T1 TSE weighted images. The size of the anterior pituitary gland, as well as the gland morphology including position of the posterior lobe T1 bright spot, were determined.

**Figure 1:**
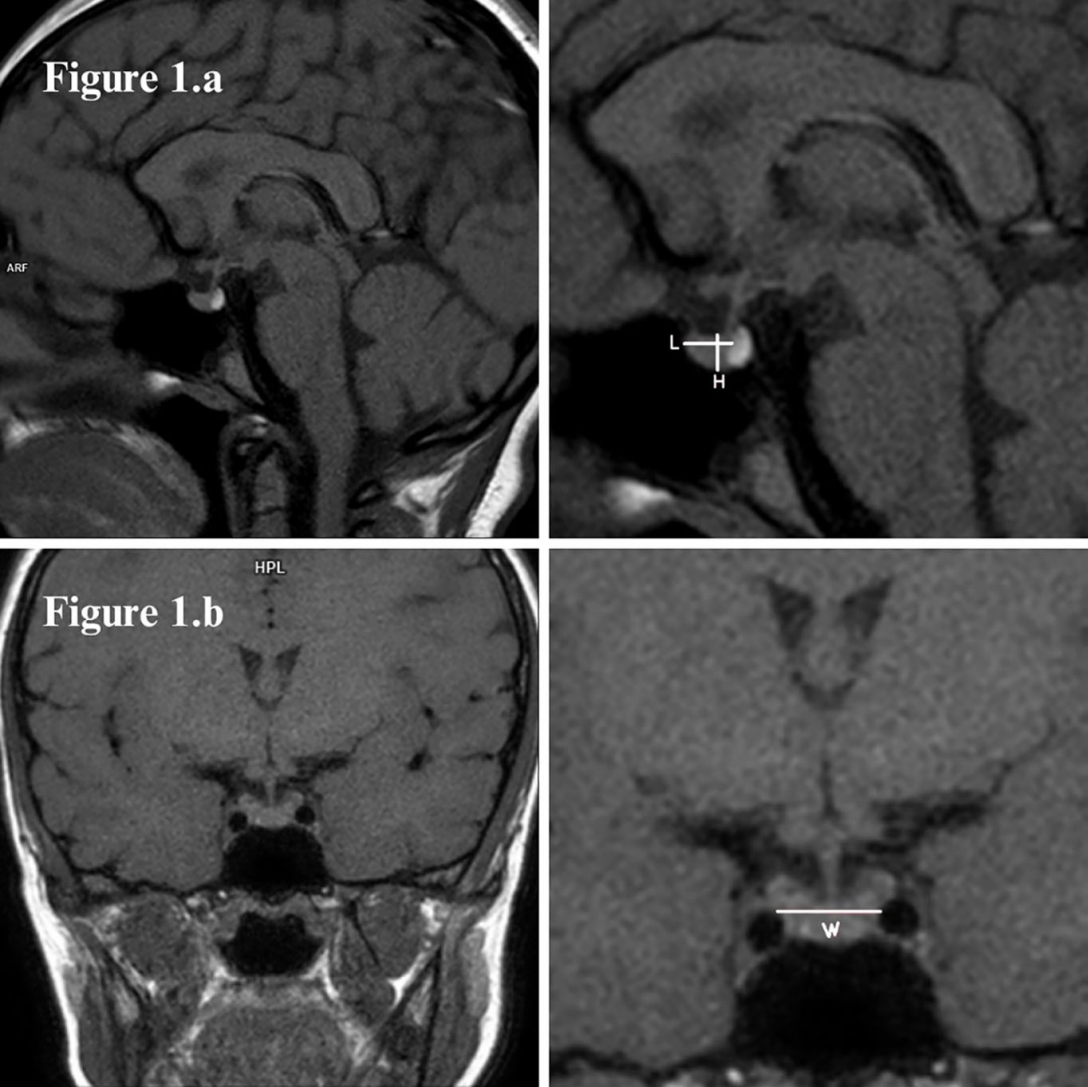
**(A)** Sagittal and **(B)** coronal T1-weighted MR images of a normal size pituitary gland, with bright spot of the posterior lobe (neurohypophysis) normally located and well-centered pituitary stalk (infundibulum). Pituitary measures were taken as shown: length (L) and height (H) in sagittal images, width (W) in coronal.

Maximal pituitary height was determined from midline sagittal images by measuring the greatest distance between the superior and inferior borders of the gland. Maximal pituitary length and width were similarly determined by measuring the greatest dimensions, the former on the sagittal images and the latter on coronal images (shown in **Figure 1**). The length of the anterior pituitary gland was measured without taking into account the neurohypophysis. Midline positioning of the image was assessed by simultaneous visualization of pituitary gland and pituitary infundibulum (stalk).

Pituitary volume was determined by the simplified ellipsoid formula: 0.5 * length * width * height. Volume measurements were adjusted in standard deviation (SD) according to age and sex by using the published data of a healthy population (9). Pituitary height was adjusted in SD according to age and sex of healthy populations published by Argyropoulou et al. (10) and Tsunoda et al. (11).

Two physicians independently analyzed the pituitary parameters to assess the reproducibility of the measurements. Both were blinded to the clinical features. The physicians were a pediatric endocrinologist (observer 1) and a pediatric radiologist (observer 2), both with extensive experience in neuroendocrinology pathology.

The criteria for defining small pituitary gland (SPG) was that used as in previously published articles (4, 5). It was defined as pituitary height or volume that are more than or equal to one SD below the reference population’s mean for age and sex. Short stature was defined as height that is more than or equal to two SD below population’s mean for age and sex.

Bi-parietal diameter (BPD) was measure by the pediatric radiologist (observer 2) to adjust a smaller head size described in FA patients (2, 3). We measured BPD on craneal images as an indicator of their head size, since data for head circumference were not available in all patients. The BPD was measured at two different levels on T2-weighted sequences on non-contrast MRI images. The BPD A measurement was taken at the level of the cerebral peduncles at the level of the red nucleus and the BPD B measurement was at the thalamic level at the level of the foramen of Monro as described by Sherafat-Kazemzadeh et al. (4). The BPDs were measured from outer cortex of one side to outer cortex of the opposite.

Abnormalities of the CNS were classified on the basis of reports made by the pediatric radiology team without taking into account pituitary gland volume measurements.

### 2.3 Statistical analysis

The intraclass correlation index (ICC) was used to determine interobserver variability and Kappa index to assess the degree of inter-observer agreement for the qualitative variable (pituitary gland shape). The ICC and kappa are a measure of reliability that varies from 0 to 1, with values closer to 1 indicating a higher concordance. This value can arbitrarily be interpreted as poor <0.40, good 0.40 - 0.75 and excellent > 0.75 - 1.00.

Each measurement was performed at least three times to the nearest millimeter by using software tools (Siemens syngo.plaza and syngo.via) and the average was used for calculations. Finally, we used the average of the two observers’ measurements to express the results.

Quantitative variables are expressed in median and interquartile range (*IQR*). Qualitative variables are expressed as absolute and relative frequencies. The Shapiro-Wilks test was used to determine whether a sample fits a normal distribution, as the sample is smaller than 30. Relationships between categorical variables were analyzed by comparing proportions using Pearson’s chi-squared test, provided there were less than 20% of cells in the crosstabulation with expected frequencies below 5. If there were more than 20% of cells with expected frequencies below 5, the two-sided Fisher’s exact test was used. The relationship between a binary exposure variable and a quantitative response was analyzed using Student’s t-test for independent samples with normal distribution. In the case of non-normality, the non-parametric Mann-Whitney U test was used for comparison, and the non-parametric Fisher-Pitman test was used for groups with fewer than 10 patients. The non-parametric Kruskal-Wallis test with Bonferroni correction was used to compare multiple means between categories when the assumption of normality and homogeneity of variances was not met. Spearman-rank correlation was performed to determine whether there were linear association between quantitative variables. Results were considered to be statistically significance with a p<0.05.

## 3 Results

**Figure 2:**
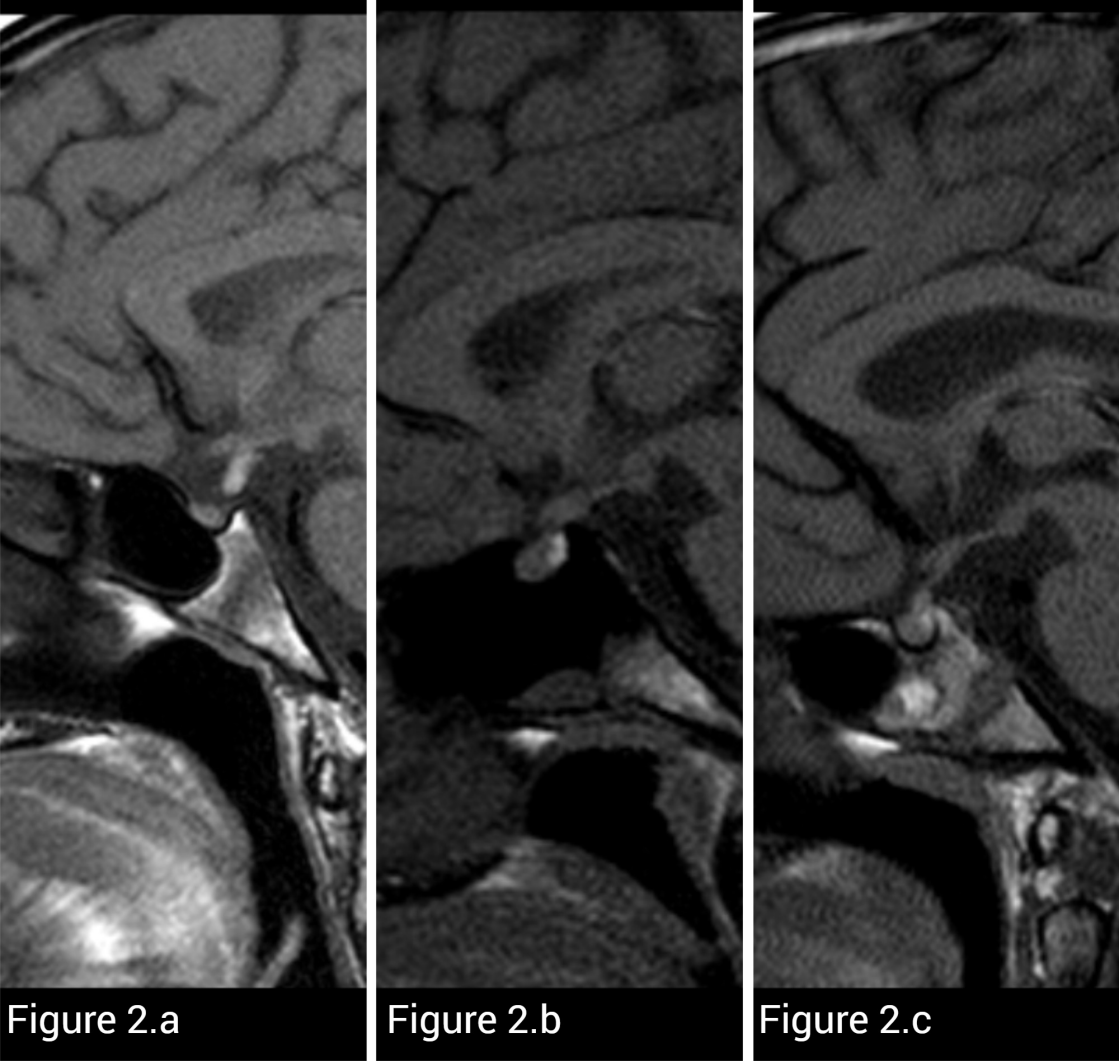
T1-weighted sagittal MR images of three different FA patients. **(A)** Small pituitary size and high signal corresponding to neurohypophysis located ectopic in pituitary stalk are noted in this patient. **(B)** MRI shows diffuse thickening of the infundibulum in another patient. **(C)** This patient presented with nodular thickening of the lower *infundibulum*. It was considered more likely than only thickening of the *pars tuberalis*.

The median age was 11.1 years (*IQR*: 8.8-14.4), with the youngest patient being 3.57 years old and the oldest 17.92 years old. There were nine males and twelve females. Eleven patients were prepubertal and 10 were pubertal. Fanconi genes mutations were FANCA (n=18), FANCD2 (n=1) and FANCG (n=2). MRI abnormalities were found in 11 patients (52.4%). Pituitary abnormalities were found in three patients [pituitary hypoplasia and ectopic neurohypophysis (shown in **Figure 2A**), mild thickening of the pituitary stalk (shown in **Figure 2B**) and mild thickening of the infundibulum (shown in **Figure 2C**)]. If we classify a pituitary volume of less than 1 SD as an abnormal MRI finding, the percentage of abnormalities in our series was 81% (17/21). Thus, it may be the most frequent anomaly followed by Chiari malformation type 1 (shown in **Table 1**).

**Table 1:** The clinical features, pituitary volume and central nervous system abnormalities of patients with Fanconi Anemia.

Table 1A. Male patients								
ENE MUTACION	TANNER	AGE (years)	HEIGHT (SD)	PITUITARY VOLUME mm^3^	PITUITARY VOLUME (SD)	HSCT	HORMONAL DEFICIENCIES	BRAIN ABNORMALITIES
**FANC-A**	**1**	6.76	-2.52	100.69	-1.31	Yes*	ND	NORMAL
**FANC-A**	**1**	8.81	-2.63	84.17	-1.81	No	ND	CHIARI 1, PLATYBASIA, VERTEBRAL ANOMALY, INTERNAL CAROTID AGENESIS
**FANC-A**	**1**	9.14	-2.28	90.34	-1.59	No	ND	NORMAL
**FANC-A**	**1**	11.30	-2.53	98.74	-1.21	Yes*	ND	THICKENING OF THE PITUITARY INFUNDIBULUM
**FANC-A**	**1**	10.73	-1.37	85.71	-1.30	No	ND	THORNWALDT’S CYST (benign choanal tumor)
**FANC-A**	**1**	14.72	-2.96	179.49	-0.01	Yes	Hypergonadotropic Hypogonadism	CORTICOSUBCORTICAL CAVERNOMAS
**FANC-A**	**3**	13.77	-1.58	133.7	-1.97	No	ND	NON-SPECIFIC WHITE MATTER LESION
**FANC-A**	**4**	15.58	-0.26	351.77	0.89	Yes*	Hypergonadotropic Hypogonadism	SUPRAVERMIAN CISTERNAL LIPOMA
**FANC-A**	**5**	17.16	-2.30	219.31	-0.36	Yes	ND	NORMAL
Table 1B. Female patients								
GENE MUTACION	TANNER	AGE (years)	HEIGHT (SD)	PITUITARY VOLUME mm^3^	PITUITARY VOLUME (SD)	HSCT	HORMONAL DEFICIENCIES	BRAIN ABNORMALITIES
**FANC-A**	1	3.57	-1,04	54.99	-2.05	No	ND	NORMAL
**FANC-A**	1	7.34	-2.07	89.77	-1.03	No	ND	NORMAL
**FANC-G**	1	7.39	-2.50	102.68	-0.79	No	ND	CHIARI 1
**FANC-A**	1	8.57	-2.96	157.71	-0.17	Yes	ND	NORMAL
**FANC-A**	1	9.59	-1.47	101.4	-1.27	No	ND	CHIARI 1
**FANC-A**	2	11.11	-3.62	226.85	0.45	Yes*	Hypergonadotropic Hypogonadism	NORMAL
**FANC-G**	3	10.50	-2.20	142.10	-0.62	Yes	ND	MILD CORTICAL ATROPHY
**FANC-A**	5	11.99	-0,63	151.21	-0.77	Yes	ND	NORMAL
**FANC-A**	5	13.82	-2.76	330.56	0.01	No	ND	NEUROGLIAL CYST
**FANC-A**	5	14.44	-1.19	222.53	-0.96	Yes	ND	MILD PITUITARY STALK THICKENING
**FANC-A**	5	16.03	-3.39	149.24	-1.17	Yes	ND	CORPUS CALLOSUM HYPOPLASIA
**FANC-D2**	5	17.92	-4.90	109.11	-2.2	Yes*	Growth hormone deficiency	SMALL ADENOHYPOPHYSIS & ECTOPIC NEUROHYPOPHYSIS

**Table 1** is divided into two parts: male patients (1.A) and female patients (1.B). * Patients who have received radiotherapy. HSCT, Hematopoietic stem cell transplantation; ND, none detected.

The median value of pituitary volume was -1.03 SD (*IQR*: -1.56, -0.36) and 133.7 mm^3^ (*IQR*: 100.69- 188.97) and sagittal height 5.05 mm (*IQR*:4.33-5.73). Sagittal height SD according to Argyropoulou et al. (10) (sagittal height A) was -0.16 SD (*IQR*: -1.12, 0.68) and sagittal height according to Tsunoda et al. (11)(sagittal height T) was -0.56 SD (*IQR*: -1.14, 0.13). A SPG was found in 52.4% (11/21) [CI95% 29.8-74.3] according to volume and 28.6% (6/21) [CI95% 11.3- 52.2] according to sagittal height A. Only three patients have a pituitary volume above 0 SD and nine patients a sagittal height A above 0 SD. Two patients had a pituitary volume less than -2 SD. One patient with a FANCA mutation had a volume of -2.05 SD and was the youngest in the cohort at 3.57 years of age, with no change in resonance or hormone deficiency at the time of the study. In contrast, the patient with the smallest volume, -2.55 SD, belonging to the FANC-D2 group, was the oldest patient in the cohort with growth hormone deficiency (**Table 1**).

There was a positive correlation between pituitary volume SD and sagittal height A SD r=0.61 (CI95% 0.23-0.83) p=0.004. All those classified as SPG by sagittal height A were consistent in small volume (p=0.012). Four out of the 15 patients who were classified as having a normal sized pituitary gland according to height A, had a small pituitary gland based on volume. In this group of patients, the median volume was -1.24 SD (*IQR*: -1.54, -1.12) and sagittal height A 0.6 SD (*IQR*: 0.05 - 1.26). No statistically significant attributable factors (type of mutation, sex, Tanner stage, height, hormone deficiency or radiotherapy treatment), were found to be associated with SPG.

Pituitary volume was larger in pubertal patients p=0.0007*. The median pituitary volume was 100.69 mm^3^ (*IQR*:89.77-103.74) in prepubertal patients versus 204.14 mm^3^ (*IQR*:142.1-226.85) in pubertal patients. There was also a positive correlation between volume and patient age r=0.61 [CI95% 0.25-0.83] p=0.0003* and tanner stage r=0.62 [CI95% 0.23-0.86] p=0.0025*. We found no correlation between pituitary height and age or pubertal stage. We also found no differences in pituitary height between sexes or pubertal status, although the pituitary height of boys was slightly lower than that of girls. The median pituitary height in boys was 4.6 mm (*IQR*: 4-5.25) and 5.12 mm (*IQR*: 4.6-5.25) in girls. There is no difference between pre-pubertal and pubertal values of the SD volume.

The ICC average for volume was 0.85 [CI95% 0.61-0.94] and sagittal height 0.88 [CI95% 0.70-0.95]. These results indicate a good‐to‐excellent correlation of measurements.

BPD A median was 130 mm (*IQR*: 125.5 - 134.5) and BPD B was 129.5 (*IQR*: 125.5-135.5). There was no difference in means between the different BPD measurements. The difference was 0.2 mm [CI95% -0.29, 0.69] p=0.4111. No differences in BPD were observed in those with or without SPG. No correlation was found between volume in mm^3^ and BPD measurements r= -0.12 [CI95% -0.54, 0.34] p= 0.6098, nor with volume SD and BPD measurements r= 0.06 [CI95% -0.39, 0.49] p= 0.7903.

Most of the patients had a straight shape (13/21) followed by a slightly convex shape (4/21) and slightly concave shape (4/21). It was described by the most experienced observer. There was no association between pituitary shape and pubertal status. The straight or concave shape was found in 7 of the pre-pubertal patients and in 10 of the pubertal patients. Of the four patients with a convex shape were females, and one was prepubertal.

In relation to the shape of the pituitary gland, the overall kappa index was 0.83 (excellent) [CI95% 0.59-1.06] p=0.0001. For straight shape classification was 0.80 [CI95% 0.53-1.07] p=0.0003, slightly convex shape was 0.83 [CI95% 0.50-1.15] p=0.0001 and slightly concave shape was 0.86 [CI95% 0.59-1.13] p=0.0001.

At the moment of the MRI the patients’ Tanner stages were: I (11/21); II (1/21); III (2/21); IV (1/21) and V (6/21). Three patients had hypergonadotropic hypogonadism at the time of the MRI and one of them had isolated growth hormone deficiency (GHD). The latter had the smallest pituitary volume, sagittal height A and B: -2.2 SD; -9.5 SD; -2.6 SD respectively. There were no significant differences between volume SD and sex, pubertal stage, or presence of endocrine deficiencies. No differences were found in pituitary height either.

The median patient height was -2.3 SD (*IQR*: -2.8, -1.5). Short stature was found in 66.7% [CI95% 43-85.4]. The patient with GHD was the shortest. Her height was 132 cm (-4.90 SD) at the age of 17.92 years old (Tanner V) when the MRI was performed. However, no statistically significant differences in height were found between patients with and without hormone deficiencies. Hormone-deficient patients were slightly shorter than those without hormone deficiency: median height -3.29 SD (*IQR*: -4.26, -1.61) vs -2.28 SD (*IQR*: -2.53, -1.47). The height SD difference is smaller if we compare non-hormone-deficient patients are compared with those with hypogonadism: median height SD -2.28 SD (*IQR*: -2.53, -1.47) vs -2.96 SD (*IQR*: -3.62, -0.26). No significant differences were found between pituitary volume and having short stature. No correlation was found between height SD and pituitary volume SD. The median body mass index (BMI) was -0.84 (IQR: -1.39, -0.15), with only 20% having a BMI ≤ 1.5 SD. There was no significant correlation between BMI and height or pituitary volume.

Sixteen patients underwent hematopoietic stem cell transplant (HSCT) and in 12 it was performed prior to MRI, with the MRI being performed a median of 5.3 years after HSCT (*IQR*: 1.0-8.2). Five of them received total body radiotherapy. There was no significant difference in pituitary volume between those who had received radiotherapy and those who had not. Although the median volume in patients who received radiotherapy is slightly lower compared to those who did not receive radiotherapy; -1.31 SD (*IQR*: -1.21, 0.45) versus 0.79 SD (*IQR*: -1.27, -0.36). No effect of having been treated with radiotherapy was found on pituitary volume SD nor on pituitary height. There was also no difference in height between patients who did or did not receive radiotherapy or HSCT. Although there were no significant differences in height, the group who received radiotherapy were slightly shorter, with a median height of -2.53 SD (IQR: -3.62, -2.52) compared with the group who did not receive radiotherapy, who had a median height of -2.25 SD (IQR: -2.80, -1.42). No differences were found between having a CNS abnormality and having received HSCT. Nor was it found with having received radiotherapy.

## 4 Discussion

Previous studies have reported that CNS abnormalities are present in 61%-90% of patients with FA, and pituitary abnormalities were the most common finding (5–7). Magnetic resonance imaging is the radiological examination method of choice for evaluating hypothalamo-pituitary region related endocrine diseases and is considered essential in the assessment of patients with suspected pituitary involvement. A simple measure of a single dimension (i.e., height) cannot be considered a totally reliable indicator of the size of a tridimensional structure such as the pituitary gland. Indeed, volume measurements could give a better picture of pituitary size; however, measurement of pituitary gland height is still the most widely used method (12). A few case reports and small population studies document abnormal pituitary glands in patients with FA based only on pituitary gland height. It is important to note that there are only a few articles in the literature indicating reference values for pituitary height in children and they use wide age ranges, which could decrease the accuracy (10, 11).

Pituitary gland size and shape change physiologically throughout life depending on age and sex. Our results show that FA patients also experience these changes, with a positive correlation between volume, age and pubertal status. Girls with FA also have a convex pituitary shape and a slightly higher pituitary height than boys. Three out of four with convex shape were in the pubertal stage, the prepubertal patient with convex shape was a girl who was 7.34 years old, so this shape could be due to proximal pubertal onset. It has been reported that girls have a slightly greater pituitary height than boys, presenting at puberty an increase in size with a convex shape (12–14). This could mean that in these patients the pituitary gland is developing similarly to the normal population, although the pituitary volume is lower than average, as only three patients have a volume above the 50th percentile. It has been proposed that this group of patients has a smaller pituitary volume that could be explained by microcephaly. However, in the study by Stivaros et al. (5) no differences were found between the BPD of controls and patients with FA and the report by Sherafat-Kazemzadeh et al. (4), despite adjusting the pituitary height for BPD, patients with FA also had a lower pituitary height than controls. We also found no correlation between BPD diameter and pituitary volume or association with SPG.

Sherafat-Kazemzadeh et al. (4) found that 7 out of 11 patients with FA to have a SPG and 8 out of 11 to have short stature, but they found no correlation between these two factors. Kanakatti Shankar et al. (8) reported that 12 out of 23 FA patients had a SPG, and they were shorter than patients with a pituitary height > 1 SD. However, they found no correlation between pituitary size and hormone deficiencies. Stivaros et al. (5) described that 13 out of 19 patients with FA had a SPG. These authors used pituitary height to classify SPG. Aksu et al. and Johnson-Tesch et al. (6, 7) reported the largest series of CNS lesions in FA, with the first indicating that seven out of 34 patients (20%) had SPG and the latter 11 out of 26 patients (42%). Johnson-Tesch et al. is the only group that described SPG based on volume compared with an age-matched control group (7). These results are consistent with those described here where 11 out of 21 had a SPG based on volume measurement; however, the number would be reduced had we used pituitary height. In our series, all those classified as SPG by pituitary height had a SPG by volume. However, four patients classified as having a normal pituitary gland according to height were classified as SPG by volume. Therefore, it appears that the pituitary volume measurement would be the best choice to assess pituitary size.

It is remarkable that there are few papers describing the CNS abnormalities in these patients, which are among the most frequent findings in FA (3). If we take into account pituitary volume equal or less than -1 SD as an abnormal MRI finding, the percentage of abnormalities in our series was 17 (81%). Stivaros et al. (5) found that 18 out of 20 (90%) had MRI abnormalities, with the majority being pituitary abnormalities. In this series, 15% presented posterior fossa abnormalities, while Chiari malformation type 1 was the most frequent as in our series (14%). Akstu et al. (6) described at least one pathological finding in the CNS in 22 out of 64 patients (65%). These authors found posterior fossa abnormalities in 8.2%, while Chiari malformation type 1 was only present in two patients. Jonhson-Tesch et al. (7) reported 61% of the patients with FA in their series had CNS abnormalities, with the most frequent abnormalities being hypoplastic clivus and hypoplastic adenohypophysis. However, they only described one patient with Chiari malformation type 1. In the general pediatric population, the incidence of Chiari I malformations is 1:100 (15), while it was found in 3 of the 20 cases of Stivaros et al. (5) and 3 out of 21 in our patients. This suggests that this is not an incidental finding and may be a frequent manifestation in FA patients. Other anomalies previously described in FA patients were also found as in our series, including: ectopic neurohypophysis, cortical atrophy, corpus callosum hypoplasia, cavernoma, platybasia, vertebral anomalies, thickened pituitary stalk, cysts and vascular anomalies (5, 6, 16–18).

Karine Sii-Felice et al. (19) studied the role of FANCA and FANCG, which are involved in the activation of the Fanconi pathway, in neural stem and progenitor cells during brain development and adult neurogenesis. They showed that FANCA- and FANCG- deficient embryos developed microcephaly due to apoptosis of proliferating neural stem and progenitor cells and the resulting decline in neuron production. Thus, abnormalities of the CNS appear to be a consequence of the effect of the FA pathway at early stages of CNS development. It is not clear whether FANC has a role in the development of the pituitary gland. Larder et al. found that FANC-A was identified in and isolated from mouse anterior pituitary gonadotrophs, suggesting that FANC-A may play a role in mediating GnRH responsiveness in mature gonadotrophs. FANC-A, FANC-C, FANC-D2, FANC-E, FANC-G mutations have been associated with pituitary stalk interruption syndrome. Brauner et al. (20) identified seven families carrying variants in genes known to be involved in FA, although only one case presented with FA disease.

Another important aspect is the verification of the veracity of the measurements through the concordance of at least two clinicians, thus ensuring the correct interpretation of the data. In our study, through the ICC which gives a composite of the consistency of measurements made by multiple observers measuring the same quantity, we found all of the results to be above 0.8, which means there is excellent correlation between observers. Therefore, the results are highly reliable.

Short stature is a common finding in FA, as between 40-60% have a short stature below the reference population, with the mean height of -2.2 SD for children being reported (21, 22). Barbus et al. (23), studied growth in 260 patients for the creation of reference charts for the FA population, finding that the 50th percentile of height-for-age in both sexes with FA corresponds to the 3rd percentile of the general population according to the 2000 Centers for Disease Control and Prevention (CDC) charts in the United States. This study found that the final height was 10 cm lower than the general population in females and 13 cm in males, with the final height in females being 153 cm (-1.65 SD) and 164 cm in males (-1.84 SD). The median patient height in our cohort was -2.3 SD, slightly lower than that described in the literature. The etiology of short stature was thought to be secondary to hormone deficiencies. However, recent studies suggest that the etiology is multifactorial and inherent to the disease (21). In support of this concept, we did not find significant differences in height between hormone-deficient and non-hormone-deficient patients, although the hormone-deficient patients had a shorter height since the GH deficient patient had an extremely pathological height (-4.90 SD). Rose et al. (22) and Wajnrajch et al. (24) reported the largest series of 120 and 54 patients respectively, and height did not differ between the GH-deficient and the GH-sufficient patients. However, other authors Giri et al. (25) presented a series of 45 patients (28 children), identifying GHD in seven patients of the whole group (15%), who were significantly shorter than those with normal GH levels. In this study, four of the seven patients had midline brain anomalies. Kanakatti Shankar et al. (8) described three patients with pituitary stalk interruption syndrome and GHD who had the lowest height of 23 patients with FA. In our cohort the patient with the lowest pituitary volume, ectopic neurohypophysis and GHD had the shortest height. Therefore, although patients with FA have short stature, it is greater in the presence of severe pituitary hypoplasia and GHD. Only two patients presented a volume lower than -2 SD, one of them being the patient with GHD. The other patient, with a volume < -2 SD, was the youngest patient. As described in the literature, patients with GHD have decreased pituitary volume compared to controls (26), therefore this patient should be followed closely since a volume lower than -2 SD could suggest a risk of developing GHD. Another possible deleterious effect on growth has been described that could be the nutritional status, but a low percentage (20%) present BMI lower than -1.5 SD as well as the published data that range between 22-38% (22, 25). We found no correlation between short stature and BMI.

One other important finding observed in this cohort is the presence of hypergonadotropic hypogonadism in three patients. All received HSCT and two received total body irradiation, but we cannot explain that HSCT is the cause because of the 12 who received HSCT, only three had this deficiency at the time of the study. Gonadal involvement may result from HSCT conditioning regimen received. Receiving body irradiation of >2 Gy and cyclophosphamide induces spermatogenesis failure and decreased ovarian function (27, 28). However, Sklavos M. et al. (29) found decreased anti-Müllerian levels regardless of having received HSCT. It has also been published that the percentage of pregnancies was similar in FA patients who had or had not received HSCT. It appears that gonadal involvement is inherent to the disease, since it has been observed that women present early ovarian failure and men present primary non-obstructive azoospermia. The importance of the FA pathway in human reproduction has been studied through mouse models (30). Ross J Hill et al. (31) showed that FANC-A knockout mice have a reduction in the number of primordial germ cells compared to wild-type littermates. All patients in this cohort with hypogonadism belong to the FANC-A group, but this has also been described in FANC-C, FANC-M, FANC-D2 and FANC-B. Therefore, it seems important to follow these patients in the long term by endocrinology team and to offer fertility counseling in FA patients (32).

Another factor to take into account in these patients that may influence both height and pituitary volume is the conditioning treatment received pre-transplant. Our study found no differences in terms of pituitary volume or height between those who had received radiotherapy prior to the MRI. They had a slightly lower volume and height, possibly influenced by the fact that the patient with GHD was in this group, considerably decreasing the mean of these values. This patient’s GHD deficiency was related to her congenital structural alteration and not to the radiotherapy treatment. The possible explanation for the unaffected pituitary volume in these patients is that the doses used in conditioning therapy are low, 1.5-3 Gy (33). Pituitary involvement occurs with higher doses (> 30 Gy) commonly used in the treatment of brain tumors. A decrease in the size of the pituitary gland has been reported with higher doses of radiation therapy (34). HSCT is known to be associated with CNS changes, mainly leukoencephalopathy and white matter injury secondary to the conditioning regimen used (35). In our cohort we did not find an association between having undergone HSCT and presenting any type of CNS anomaly. Although no association was found, it would be interesting to evaluate the performance of MRI prior to HSTC in this group of patients to clearly document the association of CNS abnormalities and FA.

### 4.1 Conclusion

Central nervous system anomalies are clearly part of the FA phenotype, with the most frequent after pituitary hypoplasia being posterior fossa abnormalities, which may have clinical repercussions in the patient. It is therefore necessary to screen them with MRI to identify those who could be candidates for neurosurgical intervention. The size of the pituitary gland is smaller in these patients but does not seem to be related to hormonal deficiencies or short stature or exposure to a low dose of total body irradiation.

## Data Availability

The original contributions presented in the study are included in the article, further inquiries can be directed to the corresponding author.
